# Novel biogenic silver nanoconjugates of *Abrus precatorius* seed extracts and their antiproliferative and antiangiogenic efficacies

**DOI:** 10.1038/s41598-023-40079-8

**Published:** 2023-08-19

**Authors:** Amritpal Kaur, Yash Sharma, Gagandeep Singh, Anoop Kumar, Nutan Kaushik, Asim Ali Khan, Kumud Bala

**Affiliations:** 1 Therapeutics and Molecular Diagnostic Lab, Centre for Medical Biotechnology, Amity Institute of Biotechnology, Amity University, Sector 125, Noida, Uttar Pradesh 201313 India; 2 Kusuma School of Biological Sciences, Indian Institute of Technology, Delhi, Hauz Khas India; 3 Section of Microbiology, Central Ayurveda Research Institute, Jhansi, CCRAS, Ministry of Ayush, Govt. of India, Jhansi, India; 4 National Institute of Biologicals, Noida, Uttar Pradesh India; 5 Amity Food and Agriculture Foundation, Amity University, Noida, Uttar Pradesh India; 6 Central Council for Research in Unani Medicine (CCRUM), Ministry of Ayush, Janakpuri, New Delhi India

**Keywords:** Biological techniques, Biological models, Mass spectrometry, Nanobiotechnology

## Abstract

Biogenic silver nanoconjugates (AgNCs), derived from medicinal plants, have been widely explored in the field of biomedicines. AgNCs for the first-time were synthesized using ethyl acetate seed extracts of *Abrus precatorius* and their antiproliferative and antiangiogenic efficacies were evaluated against cervical and oral carcinoma. Ultraviolet–Visible spectrophotometry, dynamic light Scattering (DLS), and scanning electron microscopy (SEM) were used for characterization of AgNCs. Antiproliferative activity was investigated using MTT, DNA fragmentation and in-vitro antioxidant enzyme activity assays. In-vivo chick chorioallantoic membrane (CAM) model was used to evaluate antiangiogenic activity. A total of 11 compounds were identified in both the extracts in GCMS analysis. The synthesized AgNCs were spherical shaped with an average size of 97.4 nm for AgAPE (Sox) and 64.3 nm for AgAPE (Mac). AgNCs possessed effective inhibition against Hep2C and KB cells. In Hep2C cells, AgAPE (Mac) revealed the highest SOD, catalase, GST activity and lower MDA content, whereas AgAPE (Sox) showed the highest GSH content. On the other hand, in KB cells, AgAPE (Sox) exhibited the higher SOD, GST activity, GSH content, and least MDA content, while AgAPE (Mac) displayed the highest levels of catalase activity. Docking analysis revealed maximum binding affinity of safrole and linoleic acid with selected targets. AgAPE (Sox), AgAPE (Mac) treatment profoundly reduced the thickness, branching, and sprouting of blood vessels in the chick embryos. This study indicates that *A. precatorius-*derived AgNCs have enhanced efficacies against cervical and oral carcinoma as well as against angiogenesis, potentially limiting tumour growth.

## Introduction

Cancer has been one of the leading causes of death globally for several decades^1^. According to the International Agency for Research on Cancer (IARC), approximately 10.0 million cancer deaths and 19.3 million new cases were reported worldwide in 2020^1^. In India, 26.7 million people were anticipated to develop cancer by 2021, and that number was projected to rise to 29.8 million by 2025^2^. Therefore, one of the most plausible strategies is to develop potent and effective antineoplastic drugs aimed at combating cancer.

Nanotechnology is nowadays one of the most widely used approaches in cancer research and has elucidated immense potential in cancer diagnosis and treatment^3^. Nanoparticles (NPs) or nanoconjugates (NCs) are nanoscale particles with a size of 1–100 nm and have demonstrated a therapeutic potential for a wide range of diseases due to their physiochemical properties and characteristics^4^. In addition, the nanoconjugate-based drug delivery system surpasses conventional drug delivery methods in terms of efficacy by: (1) increasing the half-life of drugs and proteins that are susceptible to degradation; (2) enhancing the solubility of hydrophobic drugs; and (3) enabling controlled and targeted drug release at the site of the disease^5^.

In recent years, nanotechnology has focussed on the development of optimal methods for the preparation of metallic NCs^6^. The green synthesis approach is regarded as the most cost-effective, long-lasting, reliable, and environmentally sustainable approaches for NC synthesis. AgNCs have been demonstrated as potent bioactive substances with extensive therapeutic applications including antibacterial, antifungal, antioxidative, antiproliferative, wound healing and anti-inflammatory activities^7–9^. AgNCs have recently been explored in anticancer therapies for HT-29^10^, MCF-7, A549^11^ and Vero cell lines^12^. Despite the many advantages of nanoconjugates as drug delivery systems, there are currently only a few nanoconjugate drugs on the market for cancer treatment, such as Doxil®, Eligard®, Abraxane®, Genexol PM®, Onivyde® etc.^5^. Therefore, research aimed at developing nanoconjugate-based drug delivery systems derived from natural resources has received considerable attention from researchers around the world to improve the efficacy of cancer therapy.

In this study, we used ethyl acetate *Abrus precatorius* Linn. seed extracts (APE (Sox)/(Mac)) as a reducing agent during green synthesis of AgNCs. *A. precatorius* belongs to the Fabaceae family and the genus *Abrus*. Its seeds and leaves possess a plethora of medicinal properties including antiproliferative and anticancer. The reported therapeutic effects were attributed to the presence of numerous bioactive compounds, including flavonoids, terpenoids, stigmasterol, and a variety of alkaloids^13,14^.

To the best of our knowledge, no report pertaining to the synthesis of biogenic silver nanoconjugates using ethyl acetate seed extract from *A. precatorius* as the reducing agent without the addition of external reducing and capping agents have been published before. We believe that employing *A. precatorius* extracts may be an effective approach for synthesizing the novel AgNCs with potential biological activities. Thus, the current study emphasized on the green biosynthesis of silver nanoconjugates using ethyl acetate seed extracts of *A. precatorius* and evaluation of their antiproliferative and antiangiogenic efficacies using in-vitro and in-vivo models.

## Results

### Chemical composition analysis

**Figure 1:**
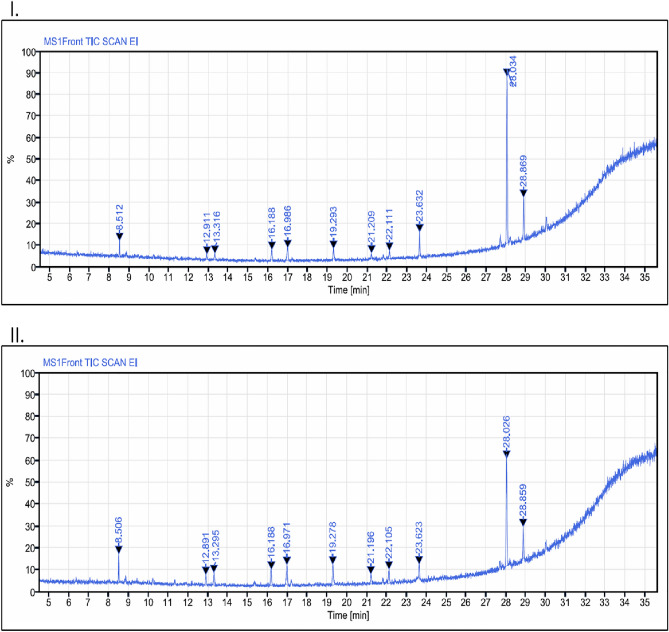
GC–MS chromatograms of *A. precatorius* seed extracts: (**A**) APE (Sox), and (**B**) APE (Mac).

GC–MS analyses of the APE (Sox) and APE (Mac) seed extracts from *A. precatorius* showed same number of compounds with different percentage concentration. A total of 11 compounds were identified in both the extracts. The seed extracts of *A. precatorius* yielded compounds largely composed of acetals, hydrocarbons, phenylpropanoids, alkanethiols, carboxylic acids, aldehydes, fatty acids, and some other essential phytochemicals. The profile of the detailed chemical composition analysis of the seed extracts of *A. precatorius* is summarized in Table 1 and Fig. 1.

**Table 1:** GC–MS identification of chemical composition for *A. precatorius* ethyl acetate seed extracts (Sox and Mac).

	Compound name^a^	MW^b^	RT^c^	Composition (%)^d^	
				APE (Sox)	APE (Mac)
1	1,1-Diethoxydecane	230.39	8.512	3.57	5.26
2	18-(1,4,7,10,13-Pentaoxacyclohexadecan-15-γl)-1,4,7,10,13,16-hexaoxacyclononadecane	510.30	12.911	1.98	3.38
3	Safrole	162.19	13.316	2.15	5.03
4	1-Undecanethiol	188.38	16.188	3.80	7.03
5	2-Propenoic acid	128.13	16.986	5.77	9.09
6	2-Tridecenal	196.33	19.293	3.57	9.11
7	1,4,7,10,13,16-Hexaoxacyclooctadecane	264.32	21.209	1.18	3.82
8	2,4-Disilapentane	100.20	22.111	2.42	4.85
9	Hexadecanoic acid	256.40	23.632	7.00	4.41
10	9-Octadecenoic acid	282.46	28.034	55.19	39.18
11	Linoleic acid	280.44	28.869	13.38	8.83

^a^Compounds listed in order of elution from a DB-Wax capillary column.

^b^Molecular weights of the identified compounds.

^c^Retention time.

^d^Relative percentage composition of the identified compounds.

### Molecular docking analysis

**Figure 2:**
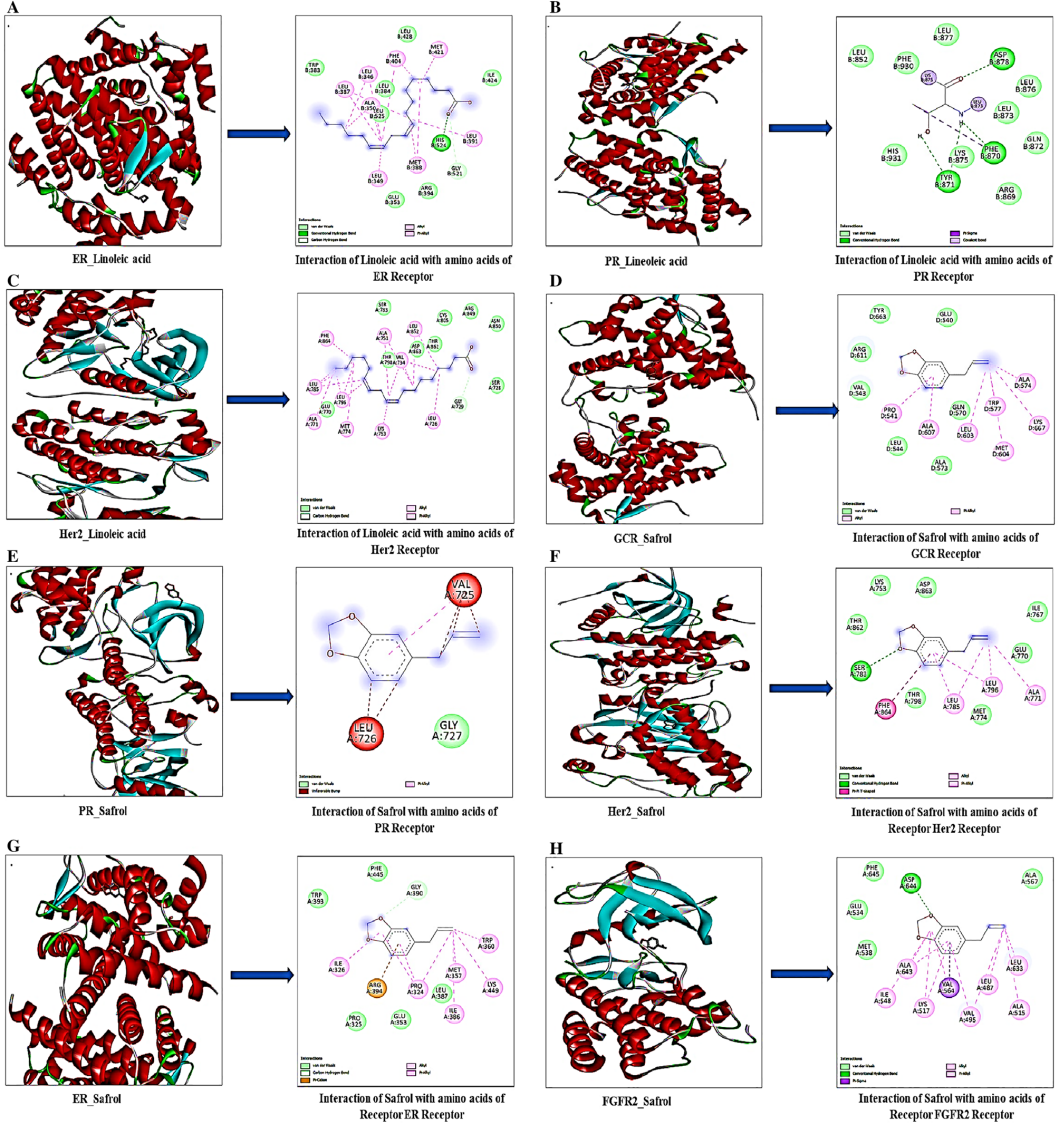
Molecular docking of linoleic acid, safrole and their interaction with amino acids, where; (**A**–**C**) linoleic acid interactions with amino acids of ER, PR, HER2 and (**D**–**H**) Safrole interactions with amino acids of GCR, PR, ER, HER2 and FGFR-2 of cervical and oral carcinoma.

The docking analysis looked at the binding affinities of the compounds identified in GC–MS against cervical and oral cancer receptors such as ER, GCR, PR, HER2, VEGF, and FGFR2. In comparison to other ligands and 5-Fluorouracil (standard), the docking analysis showed that safrole had the highest binding affinity for HER2, GCR, FGFR2, PR and ER. Similarly, linoleic acid had the highest binding affinity for the HER2, PR, and ER (Table 2, Fig. 2). Using the Discovery Studio 2021 client, the docked complexes were further visualised for their molecular interactions as illustrated in Fig. 2.

**Table 2:** Estimated ΔG (Kcal/mol) of GC–MS identified compounds against cervical and oral cancer targets.

Estimated ΔG (Kcal/mol)							
GC–MS identified compounds		Targets (receptors)					
Sr. no.	Ligands	ER	GCR	HER2	PR	VEGF	FGFR2
1	1-1-Diethoxydecane	− 5.3	− 5.2	− 4.2	− 5.5	− 4.7	− 5.1
2	Safrole	− 6.0	− 6.2	− 7.6	− 6.2	− 5.5	− 6.2
3	1-Undecanethiol	− 4.7	− 4.3	− 4.0	− 4.8	− 3.7	− 4.3
4	2-Propenoic acid	− 3.9	− 3.4	− 4.0	− 3.8	− 3.7	− 4.0
5	Linoleic acid	− 6.2	− 5.5	− 7.0	− 6.3	− 4.8	− 5.0
6	Hexadecanoic acid	− 5.5	− 5.6	− 4.7	− 5.6	− 4.0	− 4.8
7	9-Octadecenoic acid	− 5.6	− 5.3	− 4.7	− 5.9	− 4.3	− 5.4
8	5-Fluorouracil (standard)	− 5.3	− 4.9	− 5.9	− 5.3	− 4.9	− 4.8

### Biogenic synthesis of silver nanoconjugates of *A. precatorius*

**Figure 3:**
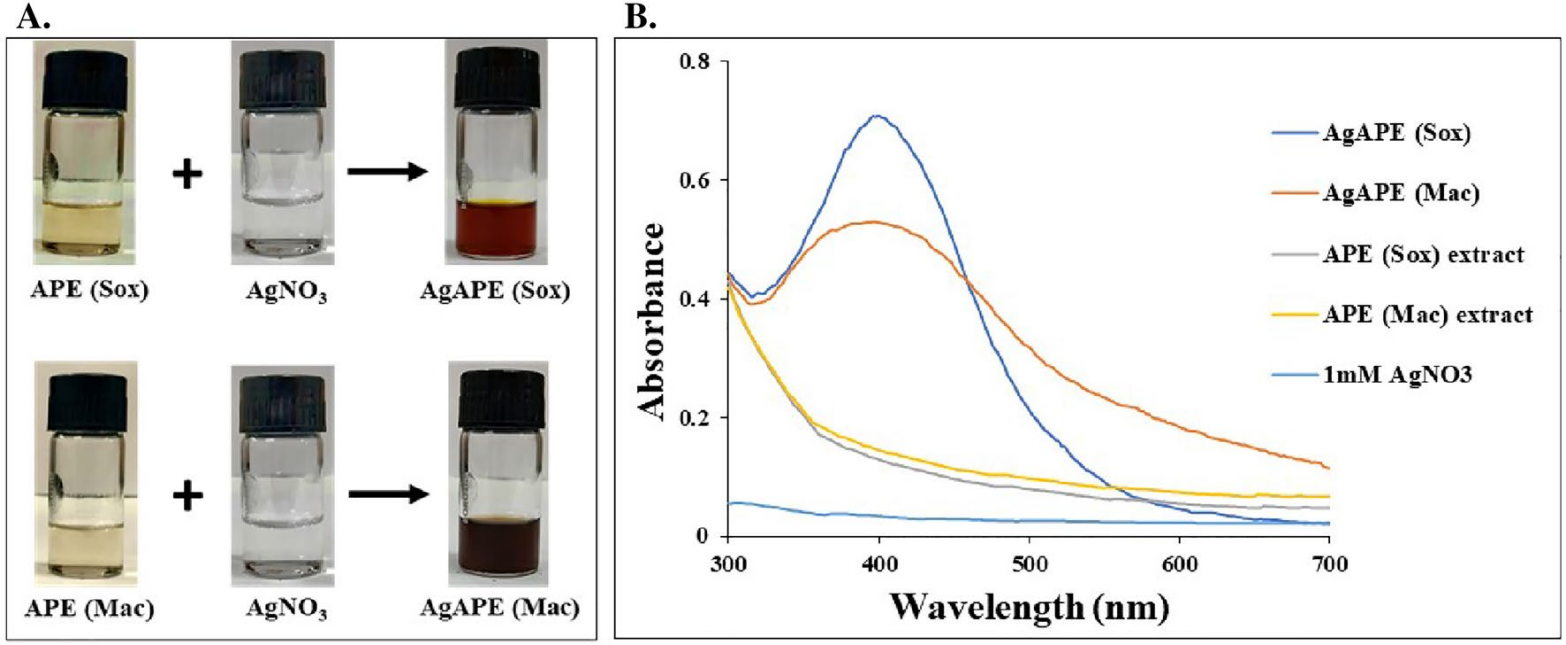
Biosynthesis of silver nanoconjugates, where (**A**) Color changes in APE (Sox) and APE (Mac) after addition of silver nitrate for nanoconjugates synthesis and (**B**) UV–Vis spectra of *A. precatorius* Sox and Mac seed extract and their green biosynthesized AgNCs along with control (AgNO_3_).

The change of color of mixture from light-green to reddish-brown and dark brown indicated the formation of AgNCs as shown in Fig. 3A. As clearly shown in the Fig. 3B, AgAPE (Sox) and AgAPE (Mac) have signature AgNCs’ absorbance peak at approximately 400 nm and 396 nm, respectively due to the surface plasmon resonance (SPR) electrons present on the nanoparticle surface. Whereas, APE (Sox), APE (Mac) and AgNO_3_ did not exhibit SPR bands.

### Effect of initial pH on the green synthesis of AgNCs

**Figure 4:**
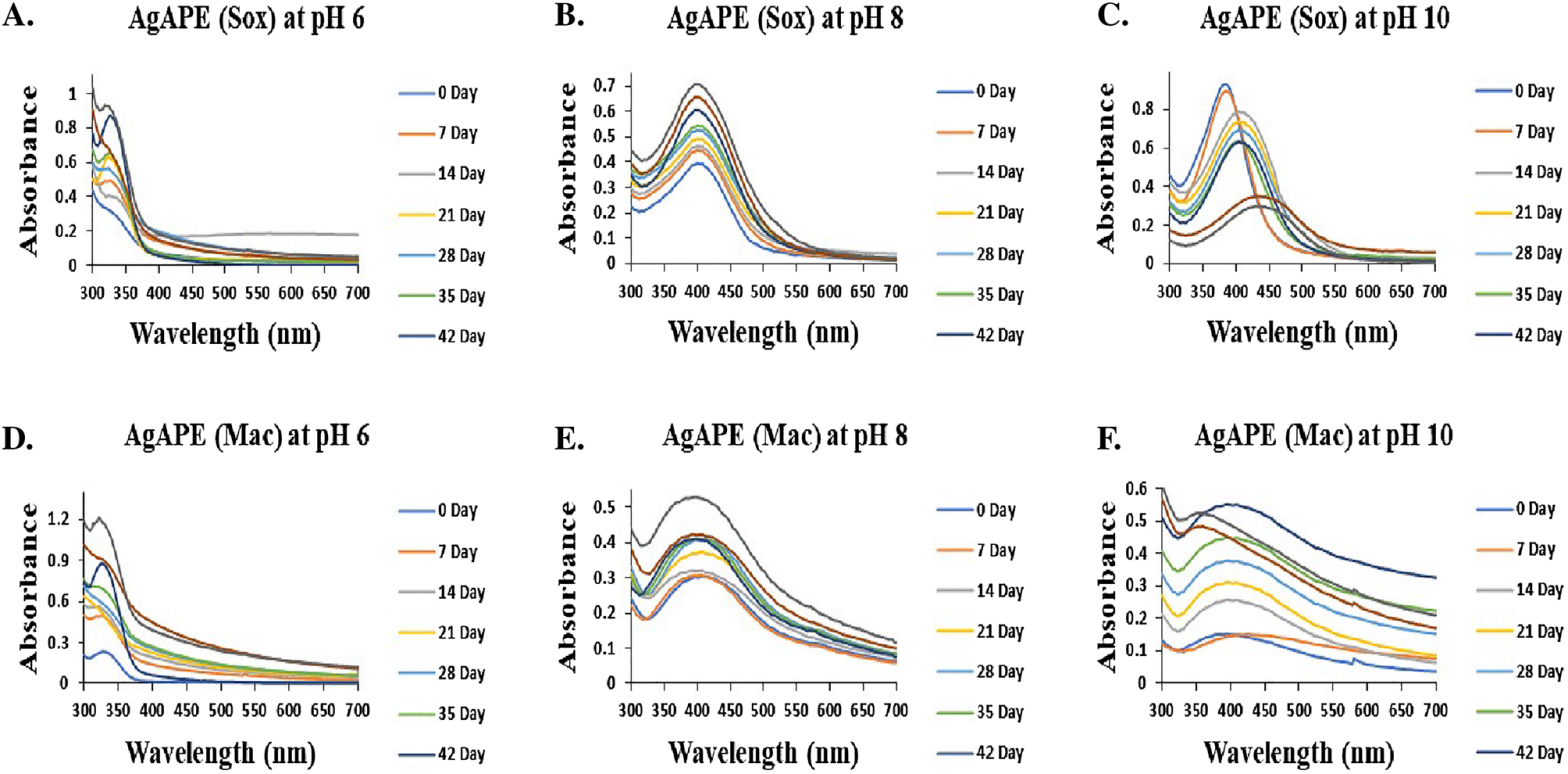
Optimization and stability analysis of AgNCs at different pH (6, 8 and 10) for 8 weeks; where, (**A**–**C**) AgAPE (Sox), and (**D**–**F**) AgAPE (Mac).

It was observed that at pH 6 (Fig. 4A,D), AgNCs exhibited a lower peak of absorption than at pH 8 and 10. However, in the AgNCs at pH 10, peak widening and a shift in wavelength was observed 7^th^ week onwards for both AgAPE (Sox) and AgAPE (Mac) (Fig. 4C,F). The characteristic absorption bands of the AgAPE (Sox) and AgAPE (Mac) at pH 8 were observed around 400 nm and 396 nm, respectively, with negligible shift in λ_max_ for the 8 weeks long study, thus found to be stable (Fig. 4B,E). The color of the reaction medium and the strength of the SPR peak of the AgNCs were investigated to be pH-dependent, showing increase in absorption with rising pH.

### Characterization of AgNCs

#### Fourier transform infrared (FT-IR) analysis

**Figure 5:**
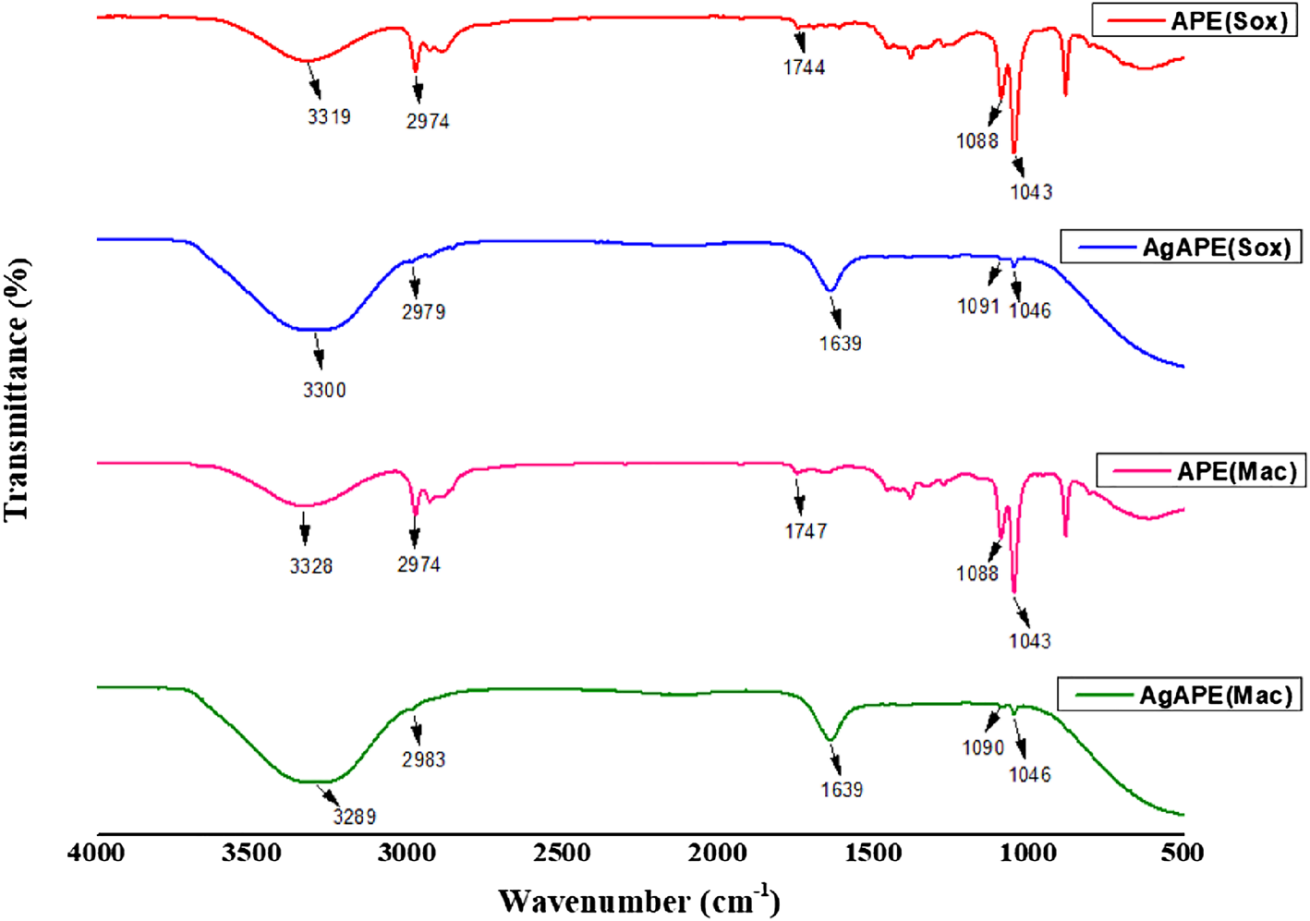
FT-IR spectra of APE (Sox), APE (Mac), AgAPE (Sox) and AgAPE (Mac).

FT-IR spectroscopy was used to identify functional groups involved in the reduction and capping of *A. precatorius* AgNCs. The FT-IR spectrum of the APE (Sox) showed major absorption peaks at 3319, 2974, 1744, 1088 and 1043 cm^−1^ and APE (Mac) showed major absorption peaks at 3328, 2974, 1747, 1088 and 1043 cm^−1^ (Fig. 5). The FTIR spectrum of AgAPE (Sox) showed major absorption peaks at 3300, 2979, 1639, 1091 and 1046 cm^–1^ and AgAPE (Mac) showed major absorption peaks at 3289, 2983, 1639, 1090 ad 1046 cm^−1^, which signify the presence of phytoconstituents that act as capping agents (Fig. 5).

#### Dynamic light-scattering and Zeta potential analysis

**Figure 6:**
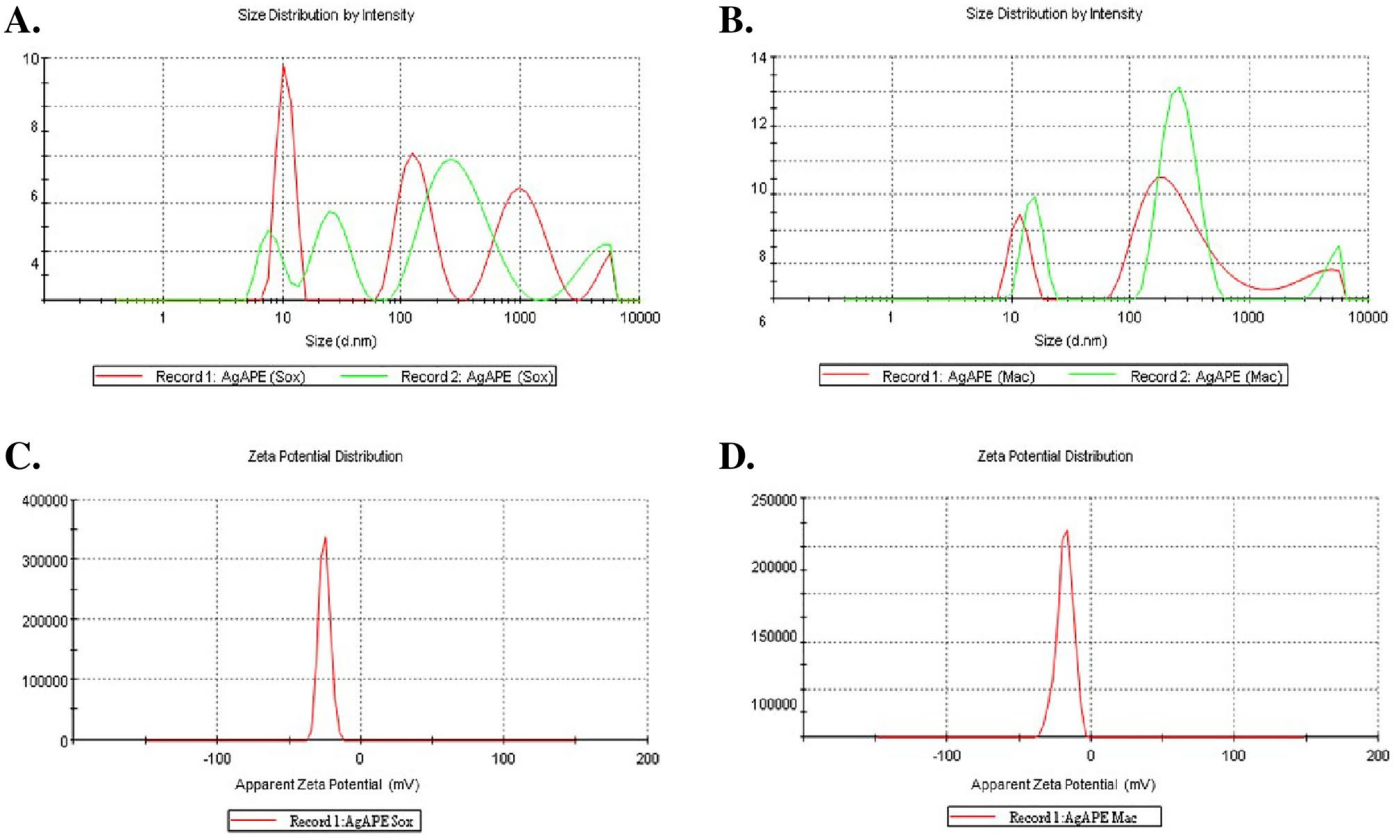
Dynamic light-scattering and Zeta potential analysis of AgNCs synthesized from *A. precatorius* seed extracts at pH 8, where, (**A**, **B**); Particle size distribution of AgAPE (Sox) and AgAPE (Mac) and (**C**, **D**); Zeta potential of AgAPE (Sox) and AgAPE (Mac).

As shown in Fig. 6A, the resulting average zeta particle size (Z-Average (d. nm)) of AgAPE (Sox) was 99.30 nm, with a polydispersity index (PDI) of 0.5 at pH 8. Similarly, the resulting average zeta particle size (Z-Average (d. nm)) of AgAPE (Mac) was 94.04 nm, with a PDI of 0.9, at pH 8, as shown in Fig. 6B. Further, the resulting average zeta potential of AgAPE (Sox) and AgAPE (Mac) were observed to be − 25.2 mV and − 18.1 mV, respectively, as shown in Fig. 6C and D.

#### Morphological analysis

**Figure 7:**
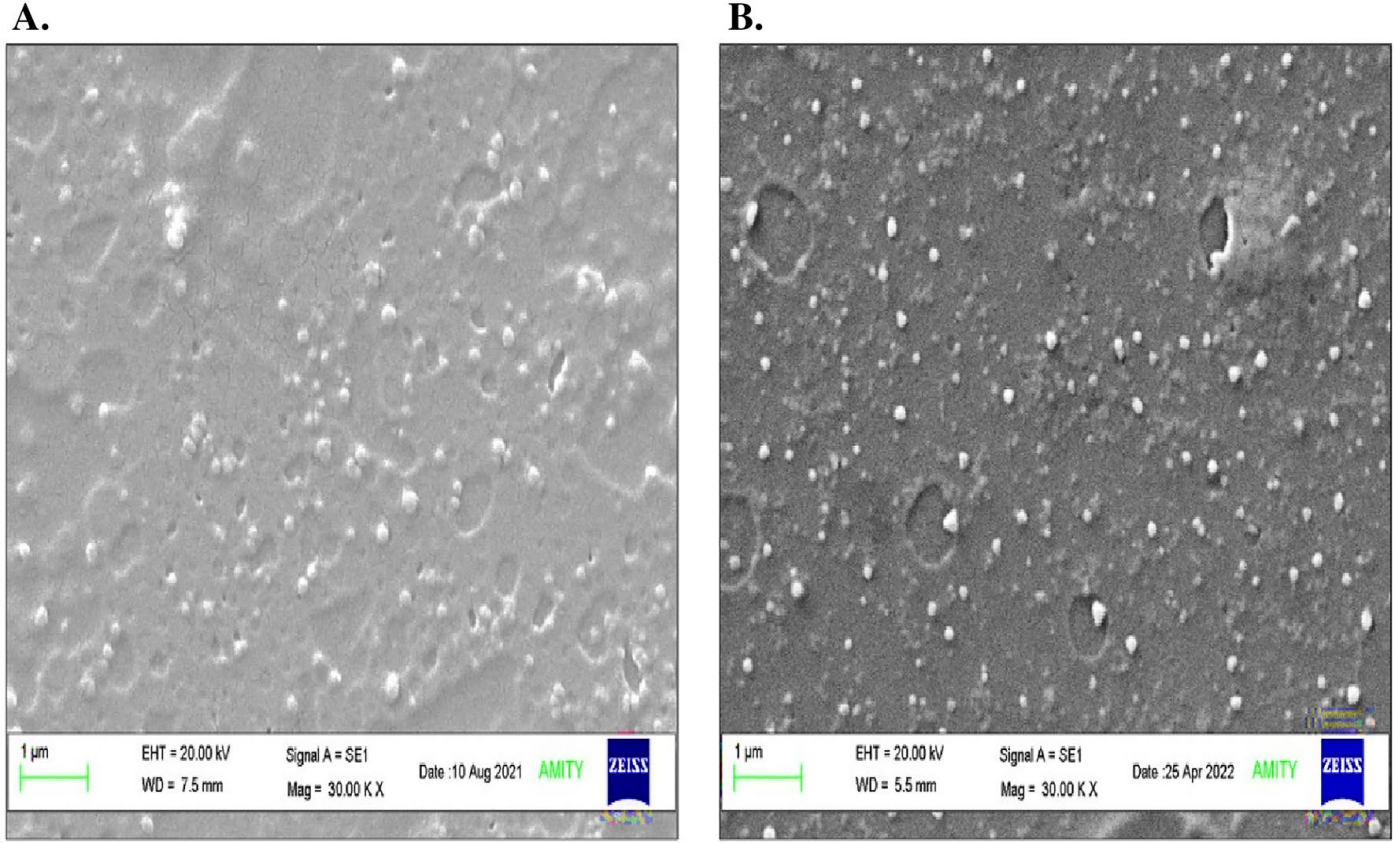
Scanning electron microscopic (SEM) analysis of AgNCs synthesized using *A. precatorius* seed extracts; where, (**A**) AgAPE (Sox) and (**B**) AgAPE (Mac).

SEM images illustrated that both biogenic AgNCs were spherical in shape. SEM analysis of biogenic AgNCs revealed distinct differences in the size of AgAPE (Sox) and AgAPE (Mac) ranging from 97.4 to 107.1 nm and 64.3 to 111.0 nm, respectively (Fig. 7A,B).

#### Elemental analysis using EDX

**Figure 8:**
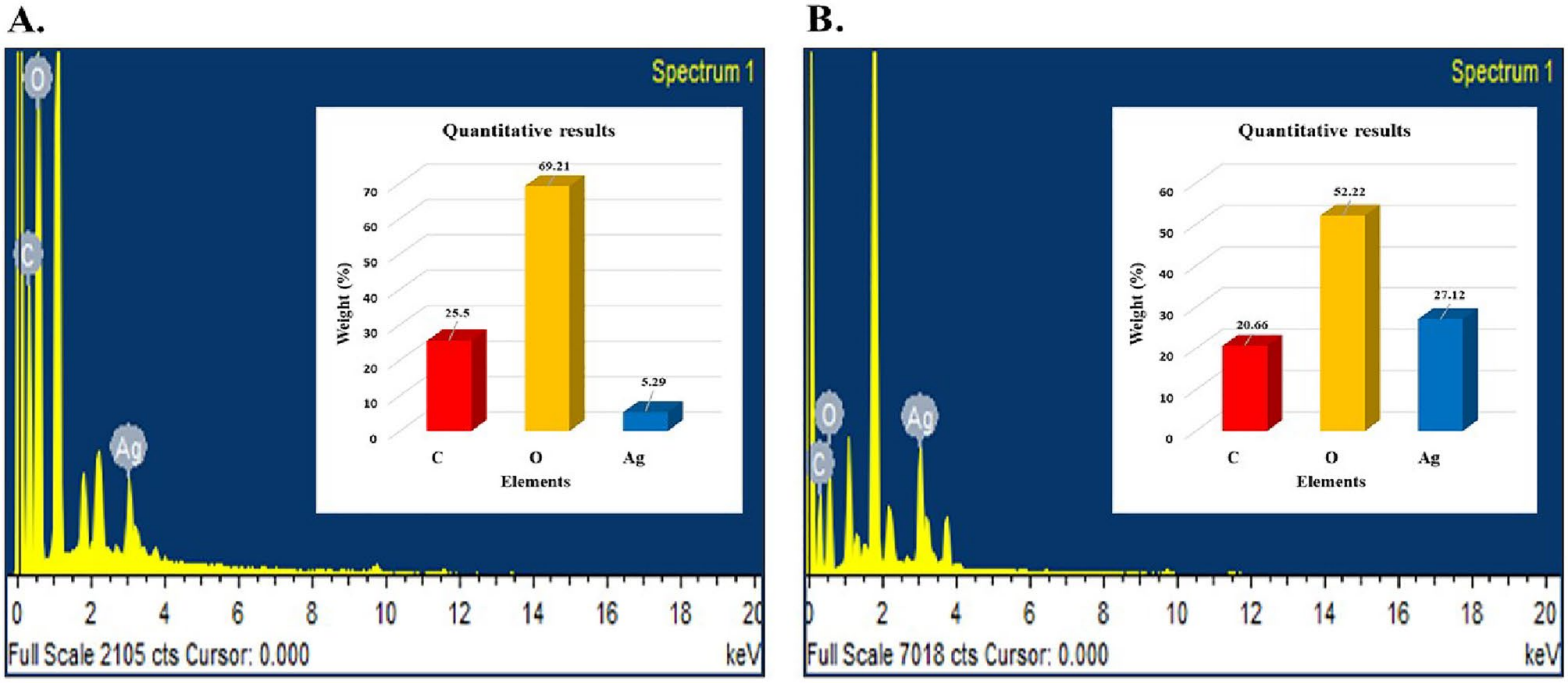
EDX spectrum of synthesized AgNCs; where, (**A**) AgAPE (Sox) and (**B**) AgAPE (Mac).

The quantitative results of EDX spectra of AgNCs showed silver yield of 5.29% and 27.12% (weight %) in AgAPE (Sox) and AgAPE (Mac), respectively (Fig. 8A,B). The absorption peaks for Ag in both the synthesized AgNCs were observed at 3 keV. On the other hand, some other signals, including carbon and oxygen were also detected in both synthesized AgNCs as shown in (Fig. 8A,B).

#### Cytotoxic activity of *A. precatorius* AgNCs against cervical and oral cancer cells

**Figure 9:**
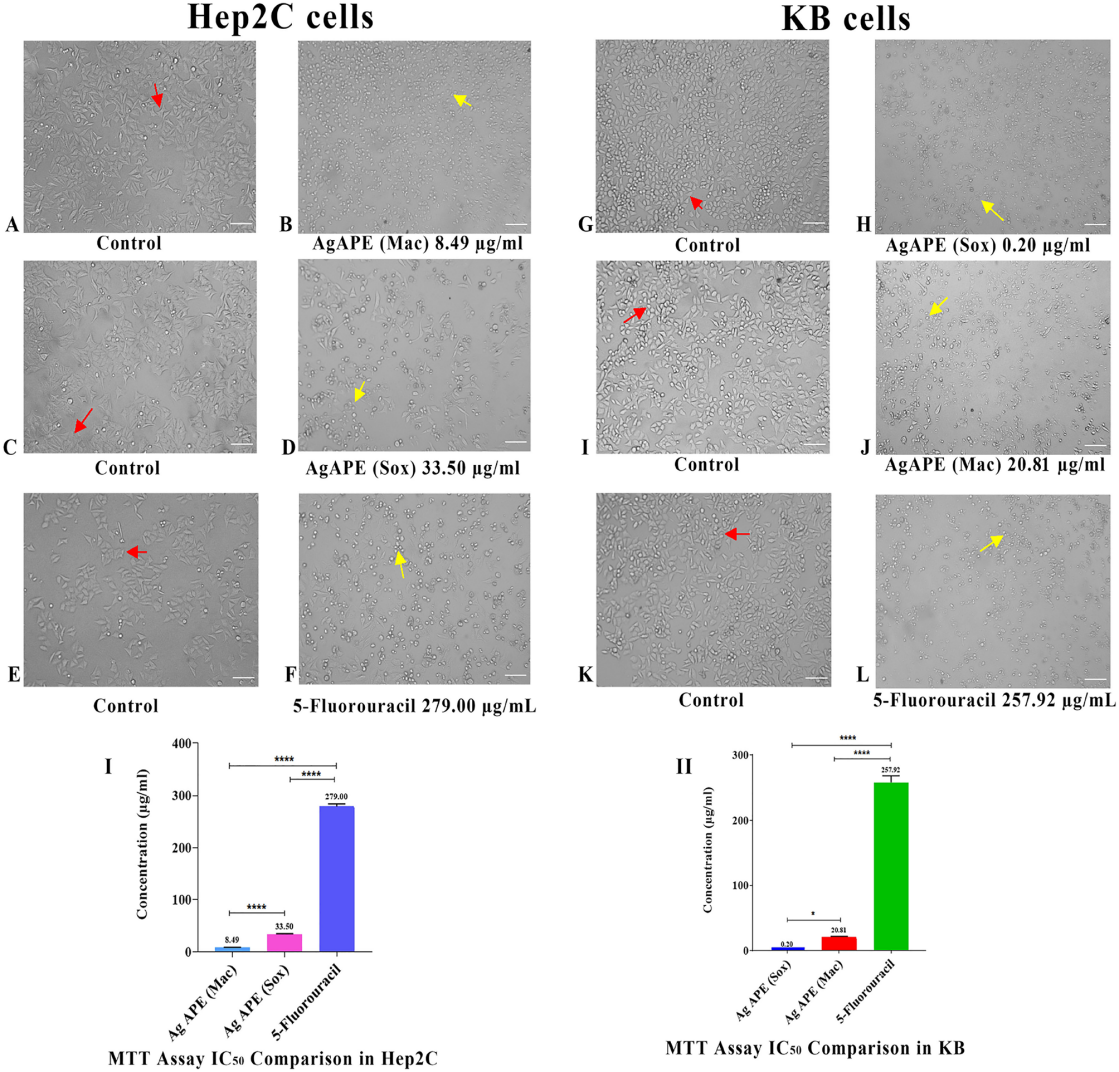
Antiproliferative efficacy of AgNCs on Hep2C and KB cells: (**I**, **II**); The comparison of average IC_50_ between AgNCs and standard (5- Fluorouracil) over 48 h. Significant p values; (*) p = 0.0114, (****) p < 0.0001 were obtained using the one-way ANOVA. (**A**–**F**) and (**G**–**L**); Morphological alterations (yellow arrow heads indicate the cells undergoing apoptosis; red arrow heads indicating live cells) in Hep2C and KB cells using IC_50_ specific values of AgNCs and standard drug after 48 h incubation. Scale bar: 100 μm.

As shown in Table 3 and Fig. 9I & (Graph 1 Supplementary), it was observed that AgAPE (Mac) had significantly higher antiproliferative activity than AgAPE (Sox) in Hep2C cells, with an IC_50_ value of 8.49 ± 0.40 µg/mL. Additionally, 5-Fluorouracil showed an IC_50_ value of 279.00 ± 4.65 µg/mL. In contrast, AgAPE (Sox) displayed significantly higher antiproliferative activity than AgAPE (Mac) in KB cells, with an IC_50_ value of 0.20 ± 0.01 µg/mL. Also, 5-Fluorouracil showed an IC_50_ value of 257.92 ± 9.98 µg/mL (Fig. 9II) and (Graph 1 Supplementary). In addition, morphological study under light microscopy showed that the untreated cells were spindle shaped while majority of the treated Hep2C and KB cells displayed cytomorphological alterations. Cell shrinkage, membrane-blebbing and nuclear fragmentation into apoptotic bodies were observed in AgNCs treated cells (Fig. 9A–L).

**Table 3:** IC_50_ values of biogenic AgNCs obtained using the antiproliferative assay.

IC_50_ (µg/mL)*		
Sample	Hep2C cells	KB cells
5-Flourouracil	279.00 ± 4.65	257.92 ± 9.98
AgAPE (Mac)	8.49 ± 0.40	20.81 ± 0.99
AgAPE (Sox)	33.50 ± 0.95	0.20 ± 0.01

*Each value is represented as a mean ± standard deviation (n = 3).

#### DNA fragmentation

**Figure 10:**
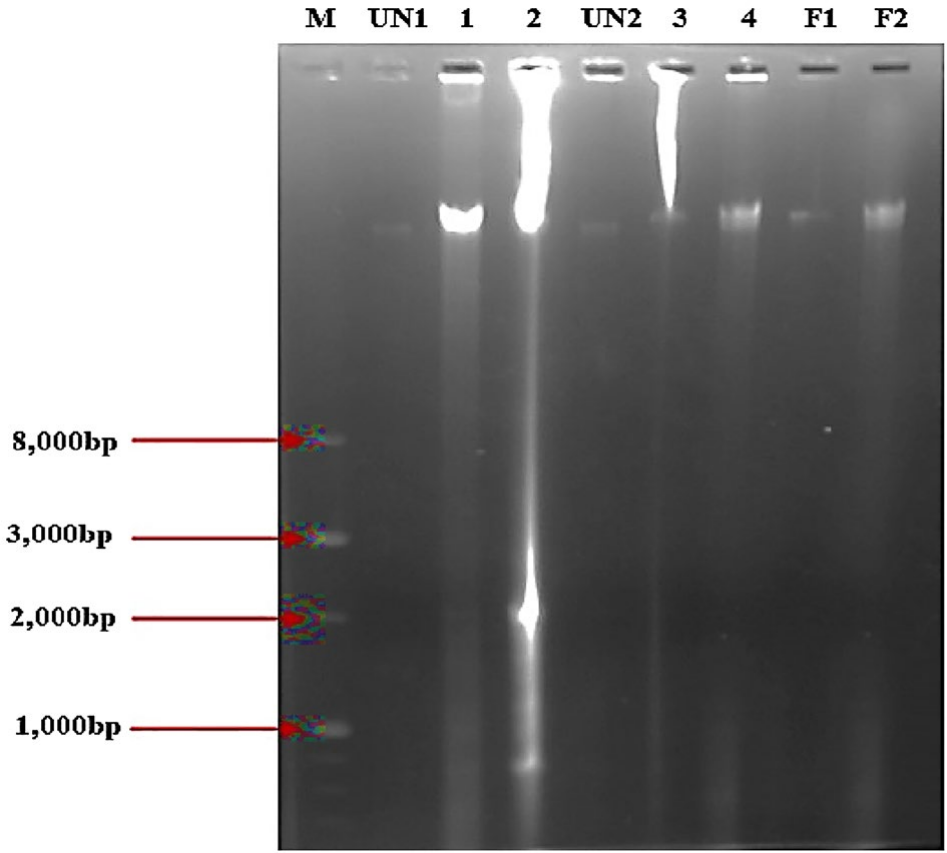
DNA fragmentation of Hep2C and KB cell lines after treated with AgNCs of *A. precatorius* at IC_50_ value for 48 h. M: DNA marker (1 kb); UN1: untreated (Hep2C cells); 1: AgAPE (Sox); 2: AgAPE (Mac); UN2: untreated (KB cells); 3: AgAPE (Sox); 4: AgAPE (Mac); F1: Positive control (5-Flourouracil) on Hep2C cells; F2: Positive control (5-Flourouracil) on KB cells.

As shown in Fig. 10, no DNA ladder pattern was observed in untreated cells. In AgAPE (Sox)/(Mac) treated cells, DNA laddering pattern (between 300 and 1000 bp) was markedly induced in both Hep2C (strong bands) and KB cells (very light bands), comparable to 5-Fluorouracil treatment.

### Antioxidant enzymes activity assay on cells

#### Superoxide dismutase activity

**Figure 11:**
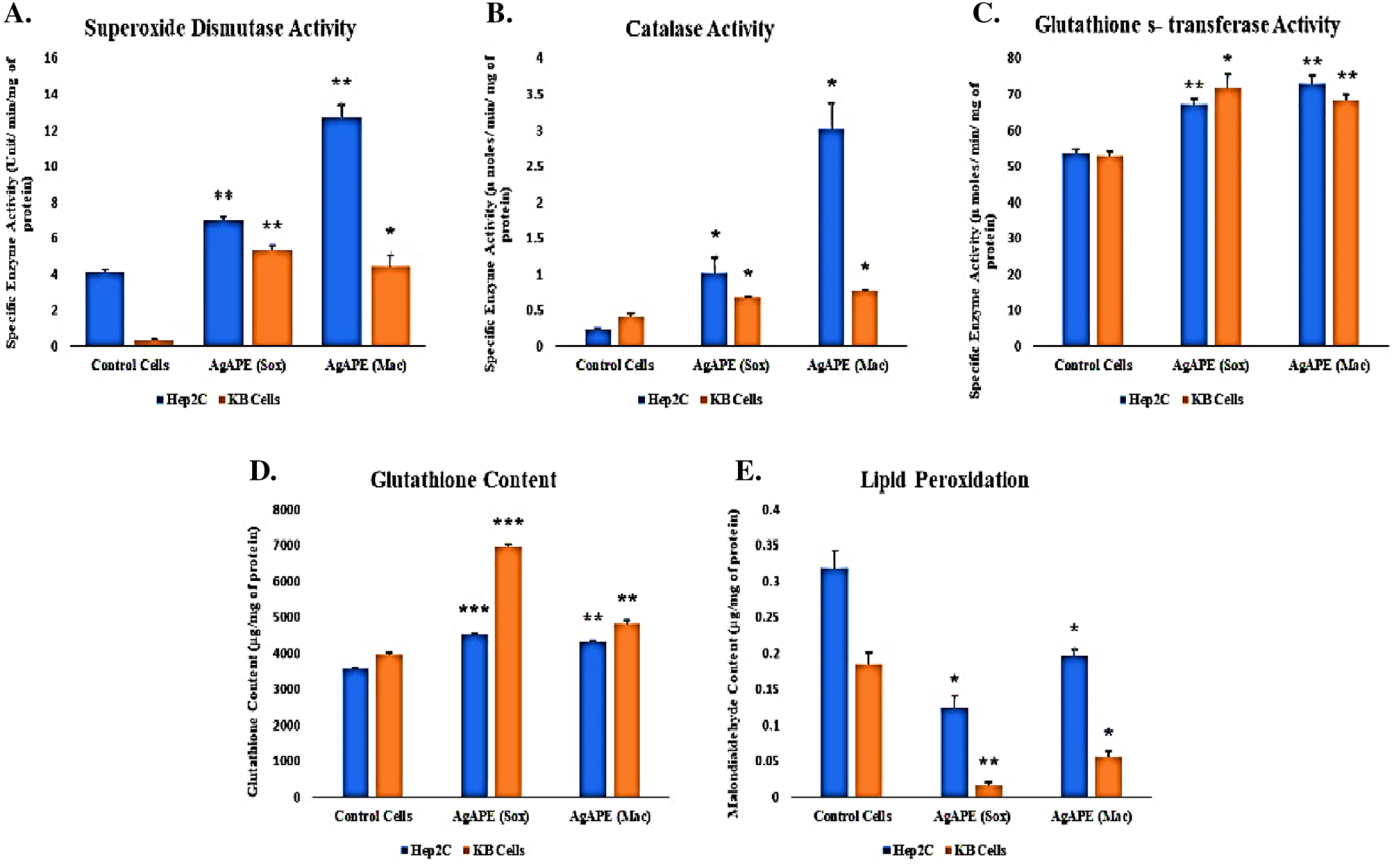
(**A**–**C**) Antioxidant enzyme activity of *A. precatorius* derived AgNCs on Hep2C and KB cells, where (**A**); SOD activity of AgAPE (Sox) and AgAPE (Mac), (**B**); CAT activity of AgAPE (Sox) and AgAPE (Mac), (**C**); GST activity of AgAPE (Sox) and AgAPE (Mac), (**D**, **E**); Non-enzyme content of *A. precatorius* derived AgNCs on Hep2C and KB cells, where (**D**); GSH content of AgAPE (Sox) and AgAPE (Mac) and (**E**); MDA content of AgAPE (Sox) and AgAPE (Mac). The represented data was obtained from three independently conducted experiments. *p ≤ 0.05, **p ≤ 0.01, ***p ≤ 0.001.

As shown in Fig. 11A, in Hep2C cells, AgAPE (Mac) had the highest SOD activity (12.71 ± 1.17 U/min/mg of protein) compared to AgAPE (Sox) (7.0 ± 0.38 U/min/mg of protein) and control cells (4.15 ± 0.20 U/min/mg of protein). Likewise, in KB Cells, AgAPE (Sox) exhibited the highest SOD activity (5.33 ± 0.42 U/min/mg of protein) as compared to AgAPE (Mac) (4.47 ± 1.0 U/min/mg of protein) and control cells (0.36 ± 0.04 U/min/mg of protein).

#### Catalase activity

In Hep2C cells, AgAPE (Mac) had the greatest catalase enzyme activity (3.02 ± 0.62 µmol/min/mg of protein) as compared to AgAPE (Sox) (1.02 ± 0.35 µmol/min/mg of protein) and control cells (0.23 ± 0.03 µmol/min/mg of protein). Furthermore, in KB cells treated with AgAPE (Mac) and AgAPE (Sox) displayed (0.76 ± 0.02 µmol/min/mg of protein) and (0.67 ± 0.04 µmol/min/mg of protein), respectively as compared to control cells i.e., 0.40 ± 0.09 µmol/min/mg of protein, as shown in Fig. 11B.

#### Glutathione S-transferase activity

In Hep2C cells, the GST activity was found to be highest upon treatment with the AgAPE (Mac) i.e., (72.88 ± 4.08 µmol/min/mg of protein) as compared to AgAPE (Sox) i.e., 67.37 ± 2.68 µmol/min/mg of protein and control cells (53.52 ± 2.40 µmol/min/mg of protein. In KB cells, treatment with AgAPE (Sox) displayed highest activity i.e., 71.91 ± 6.65 µmol/min/mg of protein compared to AgAPE (Mac) (68.64 ± 2.36 µmol/min/mg of protein) and control cells i.e., 52.95 ± 1.92 µmol/min/mg of protein as shown in Fig. 11C.

#### Glutathione content

Following quantitative analysis, we discovered that Hep2C treated cells with AgAPE (Sox) displayed the highest glutathione content, at 4539.70 ± 35.34 µg/mg of protein as compared to AgAPE (Mac), which had glutathione content of 4316.56 ± 51.52 µg/mg of protein, and control cells (3579.56 ± 41.89 µg/mg of protein). Contrarily, KB cells treated with AgAPE (Sox) had the highest GSH content (6973.74 ± 109.13 µg/mg of protein) compared to AgAPE (Mac) (4841.34 ± 136.18 µg/mg of protein), and control cells (3974.65 ± 66.03 µg/mg of protein) as shown in Fig. 11D.

#### Lipid peroxidation

In Hep2C cells, exposure to AgAPE (Sox) showed a marked decrease in the MDA content (0.12 ± 0.02 µg/mg of protein), as compared to AgAPE (Mac) (0.19 ± 0.01 µg/mg of protein), and control cells (0.31 ± 0.04 µg/mg of protein), respectively. In contrast, the MDA content in KB cells was found to be lower upon treatment with AgAPE (Sox) i.e., 0.01 ± 0.01 µg/mg of protein, whereas, AgAPE (Mac) treated cells, that showed an MDA content upto (0.05 ± 0.01 µg/mg of protein) as compared to untreated (control) i.e., 0.18 ± 0.03 µg/mg of protein (Fig. 11E).

#### In-vivo antiangiogenic activity test using CAM assay

**Figure 12:**
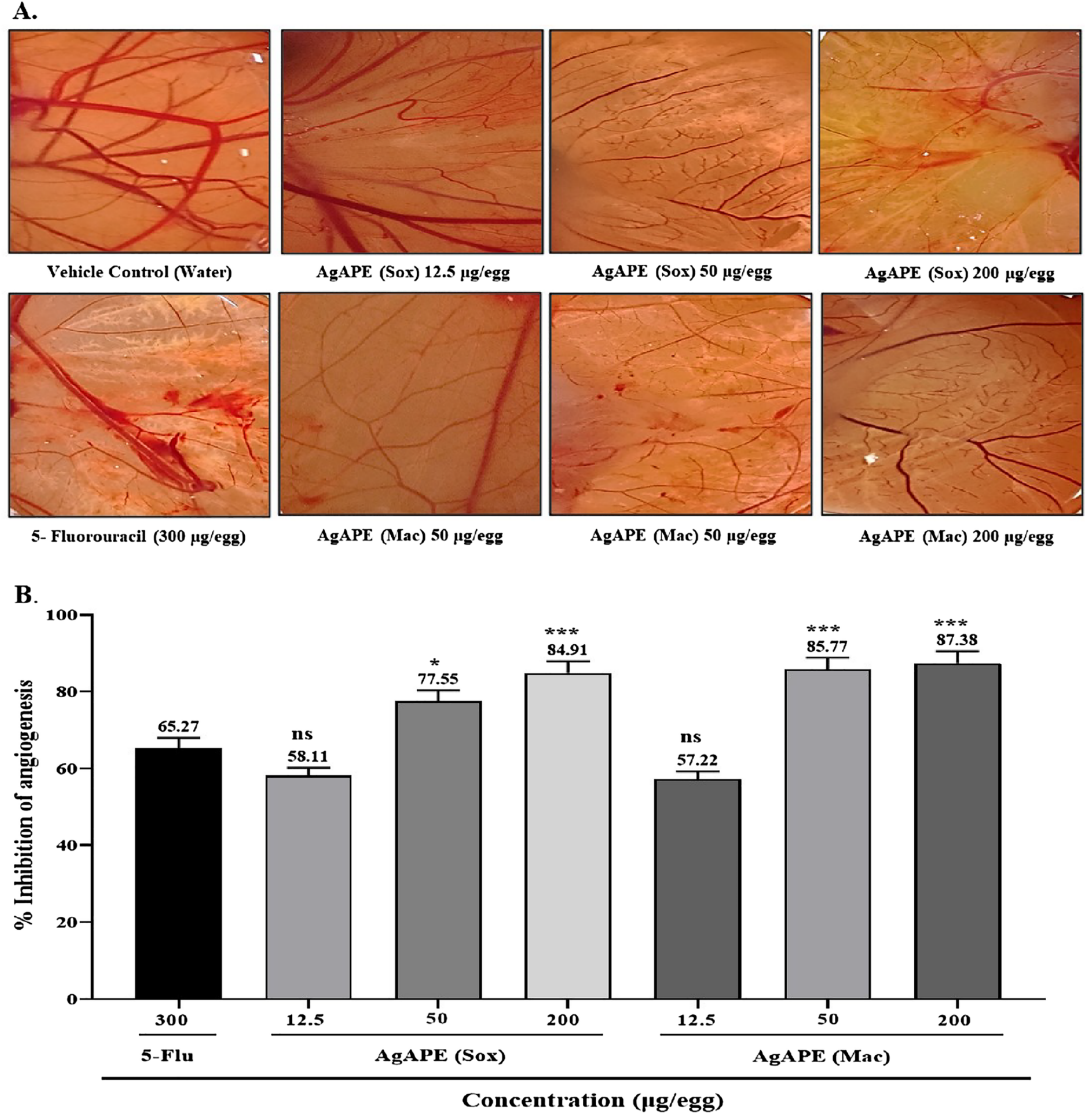
(**A**) Morphological images of CAMs after treatment with vehicle control (water), AgNCs and 5-Fluorouracil, (**B**) Percentage inhibition of angiogenesis upon treatment with 5-Fluorouracil (Standard) and AgNCs in dose dependent manner. Values are expressed as Average percentage inhibition (n = 3). *p ≤ 0.05, ***p ≤ 0.001, ns: non-significant vs. 5-Fluorouracil.

The ability of AgAPE (Sox), AgAPE (Mac) and 5-Flourouracil to inhibit the angiogenesis in- vivo was determined using the CAM assay. Water was used as the vehicle control. Prominent neovascularization was observed in treatment with water (vehicle control). 5-Fluorouracil (300 µg/egg) was used as the standard drug, exhibiting 65.27% inhibition of angiogenesis. As shown in Fig. 12A, groups treated with increasing AgAPE (Sox) concentrations (12.5, 50 and 200 µg/egg) displayed a dose dependent inhibition of chorioallantoic vessels formation by 58.11, 77.55 and 84.91%, while AgAPE (Mac) displayed inhibition of chorioallantoic vessels formation by 57.22, 85.77 and 87.38%, respectively (Fig. 12B). A significant blockade of the angiogenesis in the CAM at the concentration of 200 µg/egg of AgAPE (Sox) and AgAPE (Mac) was observed as compared to the 5-Fluorouracil (standard) suggesting that AgNCs were capable of restraining angiogenesis in-vivo.

## Discussion

Cancer continues to pose a threat for the global health due to a lack of effective pharmacological interventions. The poor efficacy of the available chemotherapeutic drugs is cited to the dissemination of malignant cells from a primary tumour to other tissues which are impeded by low delivery and high toxicity^15^. Targeted therapy has been developed as a result of the shortcomings of the already available chemotherapeutic drugs to enhance drug delivery and reduce adverse effects. Nanotechnology-based approaches such as metal nanoconjugates based drugs can overcome these limitations and play an important role in targeted therapy^16^. Green-biosynthesized AgNCs, have demonstrated promising potency for biomedical applications in recent decades. AgNCs are known for their potent antimicrobial properties, efficient inhibition of tumour cell proliferation, potent anti-inflammatory, and wound healing effects^17^. Keeping all the foregoing in mind, the current study was designed to synthesise *A. precatorius* derived biogenic AgNCs of ethyl acetate seed extracts and evaluate their in-vitro anti-proliferative, antioxidant enzyme activity, and in-vivo anti-angiogenesis activity using CAM model. Further, we assessed the binding affinity of compounds identified through GC–MS analysis against receptors mediating signalling pathways in cervical and oral carcinoma.

Preparation of plant extract is prior and most crucial step for the extraction of phytoconstituents that serves as reducing and capping agents for AgNCs^18^. Extraction of phytoconstituents is highly influenced by the type of extraction process, type of solvent, part of the plant, and temperature, might influence the yield and extraction of compounds^19,20^. In the present study, soxhlet and maceration extraction was employed to prepare the seed extracts of *A. precatorius.* Literature cites that different compounds are extracted using different extraction methods^21^. Interestingly, in current study FT-IR results showed the same functional groups in both the extracts. Similarly, based on GC–MS analyses, identical phytochemicals were found to exist in different percentage concentrations in both the APE (Sox) and APE (Mac) seed extracts of *A. precatorius* (Table 1 and Fig. 1). Based on the peak area, the most dominant compound in the APE (Sox) and APE (Mac) seed extracts was 9-Octadecenoic acid with 55.19% and 39.18%, respectively. A prior study on methanolic seed extract *A. precatorius* has reported an abundance of 7.42% of 9-Octadecenoic acid^17^, which possesses several biological activities including anticancer properties^22^. In addition, linoleic acid (13.38%) was the second most prevailing compound in *A. precatorius* APE (Sox) extract in comparison to previous study on ethanolic seed extract (1.42%)^23^. Linoleic acid possesses anti-tumour and anticancer activities thereby supressing cancer cell growth by inducing oxidant stress and mitochondrial dysfunction^24^. However, the second most prevailing compound in APE (Mac) was 2-Tridecenal (9.11%), being reported herein for the very first time in *A. precatorius* ethyl acetate seed extract. Literature cites that it possesses flavouring and aromatic properties^25^. In a previous study, 20 compounds were identified in methanolic seed extract of *A. precatorius*^17^; out of these, three (hexadecanoic acid, 9-octadecanoic acid and linoleic acid) were detected in our extracts which are considered as potential phytochemicals known for their antioxidants, anticancer, antiviral, anti-inflammatory, antiandrogenic and hepatoprotective activities^22,26^. Interestingly, this is the first report on *A. precatorius* seed extracts where safrole was also identified upto 2.15 and 5.03% in APE (Sox) and APE (Mac) respectively. Safrole is well-known for its potential antioxidant, antidiabetic, antibacterial, antifungal, and anticancer activities^27^.

The identified phytochemicals may be explored as potential drug candidates that can effectively inhibit targets at receptors sites (GCR, HER2, VEGF, FGFR2 and ER & PR) associated to the development and progression of cervical and oral cancer^28–32^. According to our *in-silico* investigation, safrole had the highest binding affinity for HER2 (− 7.6 kcal/mol), followed by linoleic acid (− 7.0 kcal/mol), when compared to the standard (5-Fluorouracil) − 5.9 kcal/mol. A previous study reported that rutin and tannic acid had the prominent binding affinities for PR, GCR, HER2, and ER^33^. Linoleic acid and 9-Octadecenoic acid had also shown promising binding affinities with ER (− 6.2 and − 5.6 kcal/mol, respectively). A recent study reported the comparable results of ER with linoleic and 9-octadecenoic acid with binding energies of − 5.5 and − 4.2 kcal/mol, respectively^34^. These findings suggest that the bioactive molecules identified from *A. precatorius* seed extracts are likely to be effective against the target proteins.

The above findings prompted us to further synthesize the biogenic AgNCs using the ethyl acetate seed extracts of *A. precatorius.* The seed extracts reduced silver nitrate (AgNO_3_) to reddish-brown and dark brown colors, indicating the formation of biogenic AgNCs. The phenomenon of surface plasmon resonance (SPR) could be attributed to the excitation and reduction of AgNO_3_^35^. Control (AgNO_3_) neither developed color nor did they display the characteristic band, indicating that reduction of AgNO_3_ did not occur under the used conditions. In addition, *A. precatorius* phytochemicals comprise hydroxyl and amino functional groups in abundance, which are known for their effective metal-reducing and capping properties, that gives AgNCs a durable coating in a single step reaction^14,33^.

Further, the synthesised AgNCs were optimized at different pH conditions and the stability of AgNCs was studied for 8 weeks. Interestingly, the absorbance peak of AgNCs rises with increasing pH, and the maximum fabrication of AgNCs occurred at pH 8, except at pH 6 and pH 10. Similarly, previous studies have also reported comparable pH effects^36,37^. Thus, we inferred that the synthesis of AgNCs is best carried out at an alkaline pH of 8, which may be due to the greater degree of ionisation that could produce powerful complexing ligands for the silver ions (48), resulting in more complex silver ion compounds. This may positively affect the ability of phytoconstituents present in *A. precatorius* extracts to cap silver ions and promote their reduction. However, in pH 6 solution, the competing biosorption process and hydroxyl protonation may be the primary factors slowing AgNCs formation and in pH 10 solution, peak widening and a shift in wavelength were observed and may have been caused by agglomeration^36,38^.

The optimized AgNCs at pH 8 were further characterized. In the current study, the observed PDI indicates the polydisperse distribution of the biogenic AgNCs. Further, zeta potential measures the strength of electrostatic charge attraction or repulsion between particles suspended in a liquid^39^. The zeta potential of the *A. precatorius* biosynthesized AgNCs was found to be negative. Due to their highly negative zeta potential, AgNCs were clearly polydisperse in nature; as a result, the electrostatic repulsive force between them prevents NCs agglomeration and greatly aids in long-term stability of solutions^40,41^.

The FT-IR spectra of the *A. precatorius* seed extracts corresponding to *A. precatorius* derived AgNCs showed major and minor shifts of the peaks reasonably due to the reduction, capping, and stabilization of the synthesized nanoconjugates^36^. A shift was observed for the peak at 3319 cm^1^ to a lower wavelength of 3300 cm^−1^ in AgAPE (Sox) and a similar shift was observed from 3328 to 3289 cm^−1^ in AgAPE (Mac) due to the involvement of the O–H or N–H stretching of phenolic compounds that are present in the seed extracts^42^. The absorption peaks at 2974 cm^-1^ in APE (Sox and Mac), 2979 cm^−1^ in AgAPE (Sox) and 2983 cm^−1^ in AgAPE (Mac) are due to the C–H stretching of the methylene group or aliphatic group^43^. The band at 1744 cm^−1^ shifted to a lower wavelength 1639 cm^−1^ in AgAPE (Sox) and band at 1737 cm^1^ to 1639 cm^1^ showing the involvement of C=C stretching of alkenes or aromatic compounds^44^. The vibrations at 1088, 1090 and 1091 cm^1^ in AgAPE (Sox and Mac), AgAPE (Mac) and AgAPE (Sox), respectively indicates the C–N stretching vibrations of aliphatic amines^45^. The band at 1046 cm^−1^ in AgAPE (Sox and Mac) ad 1043 cm^−1^ in APE (Sox ad Mac) showed the O–H stretching of the phenol groups^44^.

Besides that, the spherical shape of the synthesised AgNCs by *A. precatorius* was confirmed by the SEM images. AgAPE (Sox) ranged in size from 97.4 to 107.1 nm, while AgAPE (Mac) ranged from 64.3 to 111.0 nm. Earlier research on *A. precatorius* leaf extract showed the formation of spherical-shaped nanoconjugates with diameters ranging from 19 to 35.4 nm^46^. The elemental composition of our synthesized AgNCs was analyzed using an energy-dispersive X-ray spectrum (EDX). Furthermore, the EDX spectroscopy provides both quantitative and qualitative details about the elements that were present in the NCs. The peak at 3 keV was observed due to the SPR of Ag, indicating the successful formation of AgNCs^47^. The EDX diffraction peaks observed for carbon and oxygen suggested that the AgNCs were successfully capped by the extracellular organics from *A. precatorius* seed extracts on the surface of the AgNCs, or in the vicinity of them^48^.

Nanoconjugates have gained popularity in cancer therapy over the last decade due to their ability to selectively bind and target cancer cells^49^. We reported here for the first time the potential antiproliferative efficacies of *A. precatorius* derived biogenic AgNCs on Hep2C and KB cells. Our results demonstrated that both the AgNCs (AgAPE (Sox)/(Mac)) exhibited antiproliferative activity in a dose-dependent manner. Interestingly, AgAPE (Mac) and AgAPE (Sox), with IC_50_ values of 8.49 ± 0.4 µg/mL and 33.50 ± 0.95 µg/mL, respectively, were more effective at inhibiting cervical cancer cells (Hep2C). Prior study on Hep2C cells suggested that the IC_50_ values for *A. precatorius* APE (Mac) and APE (Sox) seed extracts were 85.91 ± 6.7 µg/mL and 142.80 ± 6.24 µg/mL, respectively^33^. This infers that the efficacy of biogenic AgNCs synthesised from ethyl acetate seed extracts of *A. precatorius* against Hep2C cells has been substantially enhanced. Similarly, in oral cancer cells (KB), AgAPE (Sox) and AgAPE (Mac) showed most promising inhibition with an IC_50_ value of 0.20 ± 0.01 µg/mL and 20.80 ± 0.99 µg/mL, respectively. A similar investigation on AgNCs from *Thuja occidentalis* extract on KB cells revealed cytotoxicity at a dose of 25 µg/mL^50^.

One major mechanism by which AgNCs are toxic to cancerous cells is through the oxidative stress caused by reactive oxygen species (ROS). The enhanced antiproliferative efficacy of AgNCs could be attributed to a synergy between AgNCs and the bioactive molecules that cap them. It is proposed that the superior cytotoxicity of AgNCs against cancerous cells may results from the higher uptake of nanoconjugates, because cancerous cells have an abnormal metabolism and a high proliferation rate, making them more vulnerable^51,52^.

Furthermore, apoptosis induction was confirmed by DNA ladder technique in AgNCs-treated cells. Apoptosis is characterized by the fragmentation of nuclear DNA into base pairs caused by the activation of endogenous nucleases. Caspase activation has been shown to promote apoptosis, activate DNase, and damage DNA^53^. In the current study, treatment with *A. precatorius* derived biogenic AgNCs resulted in DNA laddering pattern (between 300 and 1000 bp). However, several research groups have noted the presence of large DNA fragments during apoptosis and hypothesised that large DNA fragmentation occurs prior to inter-nucleosomal DNA cleavage, with these large fragments possibly acting as precursors for this cleavage^53,54^.

Moreover, genetic damage and toxicity occurs in the body due to oxidative stress which alters the expression of antioxidant enzymes in cancer cells^55^. A key strategy for boosting chemotherapy is to target the redox status of cancer cells. Several antioxidant enzymes, including GST, CAT, and SOD, neutralise the intracellular ROS. Nevertheless, there are instances when antioxidant defence mechanisms are inefficient to prevent a redox balance, which promotes the levels of ROS^56,57^. Our findings showed that, in Hep2C cells, AgAPE (Mac) revealed the highest SOD, catalase, GST activity and lower MDA content, whereas AgAPE (Sox) showed the highest GSH content. Whereas, in KB cells, AgAPE (Sox) exhibited the higher SOD, GST activity, GSH content, and least MDA content, while AgAPE (Mac) displayed the highest levels of CAT activity. Previous research found that *A. precatorius* seed extracts comprising polyphenolic flavonoids had in-vitro antioxidant enzyme activity on Hep2C and HeLa cells^33^. However, no previous research on the antioxidant enzyme activity of *A. precatorius-*derived AgNCs have been tested on cervical and oral cancer cells, to the best of our knowledge. These outcomes point to the possibility that specific interactions with the intricate cascade of cellular redox processes, including enhanced antioxidant enzyme activity and declining MDA levels in cervical and oral cancer cells, may result in a balanced antioxidant defence system and show significant antiproliferative activity.

Angiogenesis is considered as one of the hallmarks of cancer, implicated in tumour growth, invasion, and metastasis. Tumour blood vessels could be potential targets in cancer therapy since they are genetically unstable and distinct from normal vessels. CAM has therefore been extensively utilized in research on angiogenesis, tumour cell invasion, and metastasis because of its highly vascularized nature, which boosts the effectiveness of tumour cell grafting, high reproducibility, ease of usage, and cost-effectiveness^58–60^. There is evidence in the literature that blood vessel growth was inhibited by biogenic silver nanoconjugates derived from *Azadirachta indica* and *Saliva officinali*s on CAM models^59,61^. Similarly, in the current study antiangiogenesis was observed following a significant reduction in the thickness, branching, and sprouting of blood vessels using biogenic AgNCs of *A. precatorius*. This suggests that the biogenic AgNCs of *A. precatorius* possess antiangiogenic properties.

## Conclusion

The current study concludes that both APE (Sox) and APE (Mac) seed extracts of *A. precatorius* comprised potential bioactive compounds, as confirmed by GC–MS analysis. Several bioactive compounds, including safrole, linoleic acid, and 9-octadecenoic acid, were identified, and these compounds could be used in the future to isolate and purify them for therapeutic purposes.

This is the first study to our knowledge with an emphasis on the synthesis of novel biogenic AgNCs from *A. precatorius* ethyl acetate seed extracts employed as reducing and capping agents. Moreover, both AgNCs considerably enhanced their antiproliferative action against Hep2C and KB cells. The strongest binding affinities of the identified bioactive compounds against the key cervical and oral cancer receptors were also highlighted in the current research, indicating that the bioactive compounds from *A. precatorius* seed extracts are likely to be effective against the target proteins. Furthermore, this is a novel finding that the biogenic AgNCs of *A. precatorius* possess antiangiogenic action.

Therefore, in accordance with the findings of this pilot study, our ongoing research focuses on the mechanism of action of these biogenic AgNCs and their application in the development of novel alternative therapeutic strategies in biomedicine, particularly for antiproliferative and antiangiogenic efficacies. Although, there are a few challenges associated with biosynthesized AgNCs, such as genotoxicity, therapeutic window, safety profile, pharmacokinetics, that should also be addressed in future studies.

## Materials and methods

### *Abrus precatorius* seed collection and authentication

*Abrus precatorius* seeds were acquired from the local public market Khari Baoli, Kucha Chelan, Chandni Chowk, Delhi in accordance with institutional, national, and international norms and legislation. The collected seeds were stored in refrigerator at 4 °C until used. Dr. Sunita Garg (Emeritus Scientist, CSIR-NISCAIR) authenticated voucher specimens preserved at the Raw Material Herbarium and Museum, Delhi (RHMD), India, under the reference number NISCAIR/RHMD/Consult/2020/3697-98-2^33^.

### Chemicals and reagents

The analytical grade chemicals were purchased from Hi-Media and Merck, India. AgNO_3_, Et–Br and standard drugs were purchased from Sigma-Aldrich, India. MTT reagent was purchased from Merck, India. Dulbecco's Modified Eagle's Medium (DMEM), Fetal bovine serum (FBS), streptomycin (2500 U/mL) and penicillin (5000 U/mL) were procured from Gibco (USA), India.

### Preparation of *A. precatorius* Ethyl acetate seed extracts

The seeds were rinsed in water to eliminate dirt and soil particles, then dried and ground into a fine powder. Ethyl acetate seed extracts were prepared using two different extraction methods: Soxhlet and maceration according to previously published methodology^33^.

The samples were named as APE (Mac) and APE (Sox); ethyl acetate extract prepared by maceration and Soxhlet method, respectively.

### Gas chromatography-mass spectrometry (GC–MS) analysis

The analysis of chemical composition of ethyl acetate seed extracts of *A. precatorius* was performed using standardized method with minor modifications^62^. The extracts (0.1 mg/mL) were analysed by Gas Chromatography-Mass Spectroscopy (GC–MS) (8860/5977, Agilent, California, United States) using a DB-WAX capillary column (30 m length × 0.25 mm ID × 0.25 μm film thickness) and using Helium (purity > 99.999%) as the carrier gas with the constant flow rate of 1.0 mL/min. The compounds were detected based on the retention time relative to the mass spectra using National Institute of Standards and Technology library for GC–MS.

### Molecular docking

Three-dimensional SDF files of 1-1-diethoxydecane, safrole, 1-undecanethiol, 2-propenoic acid, linoleic acid, hexadecanoic acid, 9-octadecanoic acid and 5-flourouracil were downloaded from PubChem database (https://pubchem.ncbi.nlm.nih.gov/). PDB files of Estrogen (PDB ID: 1ERR), Glucocorticoid (PDB ID: 1M2Z), HER 2 (PDB ID: 3PP0), Progesterone (PDB ID: 1SQN), VEGF (PDB ID: 1FLT) and FGFR 2 (PDB ID: 2PVF) were downloaded from Protein data bank (https://www.rcsb.org). Molecular docking of the identified compounds with selected drug targets was performed using Autodock Vina employing previously published methodology^33^. Discovery studio 2021 was used to observe the interaction of amino acids with molecules.

### Green synthesis of silver nanoconjugates

An aqueous solution (1 mM) of silver nitrate (AgNO_3_) and 10 mg/mL ethyl acetate seed extracts were prepared separately. 1 mL of each extract was added to 9 mL of AgNO_3_ solution (1 mM) and stirred at 200 rpm for 60 min in dark place at room temperature^18^. The reaction mixture was adjusted to alkaline pH 8, which was determined as the optimum pH by initially adjusting the reaction mixtures from pH 6 to pH 10 using 1.0 M NaOH solution. The reaction mixtures were centrifuged at 12,000 rpm for 20 min to obtain the AgNCs^63,64^.

The prepared AgNCs were named as AgAPE (Sox); silver nanoconjugate of ethyl acetate extract obtained by Soxhlet, AgAPE (Mac); silver nanoconjugate of ethyl acetate extract obtained by Maceration.

### Characterization of silver nanoconjugates

UV–Vis spectra were recorded to check the reduction of silver nitrate with *A. precatorius* ethyl acetate extract using a Systronics UV–Vis Spectrophotometer in the range of 300–700 nm. A Zetasizer nano ZS (Malvern Instruments, Malvern) was used to determine the nanoconjugates' average hydrodynamic particle size distribution and zeta-potential. In order to prepare a well-dispersed suspension, the sample was first diluted with MilliQ water followed by 10 min of ultrasonication. FT-IR spectrophotometer (PerkinElmer, Frontier ATR/IR) was used to compare the IR spectra of both *A. precatorius* seed extracts and AgNCs in the λ range of 4000–500 cm^−1^. The structural morphology and elemental analysis of the AgNCs was studied using ZEISS EVO Scanning Electron Microscope and Energy Dispersive X-Ray Spectroscopy (EDX)^65,66^.

### Antiproliferative activity

Antiproliferative activity of *A. precatorius* derived AgNCs were evaluated using 3-(4, 5-dimethylthiazol-2-yl)-2, 5-diphenyltetrazolium (MTT) assay on Hep2C and KB cells according to the standardized protocol^33^. Around 1 × 10^4^ cells/well were seeded into flat-bottomed 96-well culture plates and incubated for 24 h at 37 °C, 5% CO_2_ and 95% air. Hep2C cells were treated with 5-Fluorouracil (12.5–400 μg/mL), AgAPE (Mac) (1.56–12.5 μg/mL) and AgAPE (Sox) (1.56–50 μg/mL)^33^. Further, KB cells were treated with 5-Fluorouracil (12.5–400 μg/mL), AgAPE (Mac) (1.56–25 μg/mL) and AgAPE (Sox) (0.03–0.31 μg/mL). 10 μl of MTT reagent (5 mg/mL) was added after 48 h of incubation, and the mixtures were then reintubated for 3 h. The resultant formazan was dissolved in DMSO (100 μL). Finally, the absorbance of formazan was measured at 570 nm using an automated microplate reader (Bio-Rad, Illinois, USA). Experiments were carried out in triplicates. The absorbance between the samples and the negative control was compared to determine the cell viability (%). IC_50_ values were also determined.

The percent inhibition was calculated by using the following formula:

$$ \% \;{\text{Loss}}\;{\text{of}}\;{\text{viability}} = { 1}00 \, - \, \left( {{\text{mean}}\;{\text{OD}}\;{\text{of}}\;{\text{test}}\;{\text{compound}}{-}{\text{mean}}\;{\text{of}}\;{\text{OD}}\;{\text{of}}\;{\text{negative}}\;{\text{control}}} \right) \, / \, \left( {{\text{mean }}\;{\text{OD}}\;{\text{of}}\;{\text{positive}}\;{\text{control}}{-}{\text{ mean}}\;{\text{OD}}\;{\text{of}}\;{\text{negative}}\;{\text{control}}} \right) \, \times { 1}00 $$

### DNA laddering assay for DNA damage analysis

In 60 mm dishes, Hep2C and KB cells were cultured and harvested after being exposed to IC_50_ doses of AgNCs and 5-Fluorouracil as a positive control. DNA extraction was then carried out using a standard phenol/chloroform extraction method^67^. TE buffer was used to dissolve the extracted DNA then it was loaded onto a 1% agarose gel and visualised with a gel doc system.

### In-vitro antioxidant enzyme activity assay

Assessment of antioxidant effect of AgAPE (Sox) and AgAPE (Mac) on Hep2C and KB cells was evaluated using the standardized protocol for estimation of enzymatic activities i.e., superoxide dismutase (SOD), catalase (CAT), Glutathione-S-Transferase (GST) and non-enzyme content i.e., Glutathione content (GSH) and lipid peroxidation (Malondialdehyde (MDA) content)^68^.

### Chick chorioallantoic membrane (CAM) assay

Using an in-vivo CAM assay, antiangiogenic action of AgNCs was evaluated. The present study used the freshly fertilized chicken eggs procured from Kegg Farms Private Limited, Gurgaon, India. Briefly, eggs were cleaned gently with 70% ethanol and incubated for 8 days at 37 °C in 85% humidity. A hypodermic needle was used to puncture a tiny hole in the shell for concealing the air sac. Candling was used to locate a second hole on the wide side of the egg, directly above the vascular region of the embryonic membrane. By injecting negative pressure via the first hole, a fake air sac was formed underneath the second hole, allowing the CAM to detach from the shell. Using sterile forceps and scissors, a window of approximately 1.0 cm^2^ was cut into the shell above the detached CAM on day 9. Thereafter, 12.5, 50 and 200 μg/egg of AgAPE (Sox) and AgAPE (Mac), while 300 µg/egg of standard (5-Flourouracil) were loaded onto a sterile filter paper disc. AgNC-treated and untreated discs were placed aseptically on growing CAMs. The treated CAM samples were placed back in the incubator after windows were sealed with sterile parafilm. Angiogenesis along the filter paper disc was photographed using iPhone 13 (Apple inc., USA) camera after 48 h of incubation at 37 °C^69^. At least 3 eggs were used per sample, and the experiment was repeated 3 times.

Digital images of the CAM sections were collected using an iPhone 13 (Apple inc.) camera. The images were then analyzed using ImageJ software. The area of vessels contained in the CAM system was measured for each sample. The resulting antiangiogenetic activity was represented as the percentage inhibition of angiogenesis as compared to the vehicle control (water) for each set of samples.

### Statistical analysis

The statistical results from three independent experiments were reported as mean ± standard deviation (SD). One-way analysis of variance (ANOVA) and the Tukey's multiple comparisons test were performed for the statistical analysis. A value of *p* ≤ 0.05 in the statistical analysis was considered as significant. GraphPad Prism version 8.0.2 was used to perform all the statistical analyses.

### Supplementary Information

Supplementary Information.

## Data Availability

All data generated or analysed during this study are included in this manuscript (and its Supplementary Information file).
