# Author Correction: Novel biogenic silver nanoconjugates of *Abrus precatorius* seed extracts and their antiproliferative and antiangiogenic efficacies

**DOI:** 10.1038/s41598-023-44725-z

**Published:** 2023-10-16

**Authors:** Amritpal Kaur, Yash Sharma, Gagandeep Singh, Anoop Kumar, Nutan Kaushik, Asim Ali Khan, Kumud Bala

**Affiliations:** 1 Therapeutics and Molecular Diagnostic Lab, Centre for Medical Biotechnology, Amity Institute of Biotechnology, Amity University, Sector 125, Noida, Uttar Pradesh 201313 India; 2 Kusuma School of Biological Sciences, Indian Institute of Technology, Delhi, Hauz Khas India; 3 Section of Microbiology, Central Ayurveda Research Institute, Jhansi, CCRAS, Ministry of Ayush, Govt. of India, Jhansi, India; 4 National Institute of Biologicals, Noida, Uttar Pradesh India; 5 Amity Food and Agriculture Foundation, Amity University, Noida, Uttar Pradesh India; 6 Central Council for Research in Unani Medicine (CCRUM), Ministry of Ayush, Janakpuri, New Delhi India

Correction to: *Scientific Reports* 10.1038/s41598-023-40079-8, published online 19 August 2023

The original version of this Article contained an error in the author list. Tapan K. Chaudhuri was incorrectly listed as an author of the original Article, and has subsequently been removed.

The Author Contributions section now reads:

“Ap.K.: Methodology, Formal analysis, Validation, Data curation, Investigation, Writing—Original Draft. Y.S.: Investigation, Visualization, Methodology, Software. G.S.: Writing—Review & Editing. A.K.: Resources, Supervision, Visualization. N.K.: Data curation, Resources. A.A.K.: Writing—Review & Editing, K.B.: Conception, Design, Methodology, Supervision, Data curation, Writing—review & editing, Project administration.”

The original Article and Supplementary Information files have been corrected.

