# Supplementary material for: Novel biogenic silver nanoconjugates of Abrus precatorius seed extracts and their antiproliferative and antiangiogenic efficacies
Source: Sci Rep. 2023 Aug 19;13:13514. doi: 10.1038/s41598-023-40079-8 (PMC10439965; doi:10.1038/s41598-023-40079-8)
Supplement: Supplementary file 1 — Supplementary Information. [file 41598_2023_40079_MOESM1_ESM.docx]

**Novel Biogenic Silver Nanoconjugates of *Abrus precatorius* Seed Extracts and Their Antiproliferative and Antiangiogenic Efficacies**

Amritpal Kaur ^a^, Yash Sharma ^a^, Gagandeep Singh ^b, c^, Anoop Kumar ^d^, Nutan Kaushik ^e^, Asim Ali Khan ^f^, Kumud Bala ^a*^­­­­­­­­­­­­­­

^a^ *Therapeutics and Molecular Diagnostic Lab, Centre for Medical Biotechnology, Amity Institute of Biotechnology, Amity University, Uttar Pradesh, India*

*^b^ Kusuma School of Biological Sciences, Indian Institute of Technology, Delhi, Hauz Khas, India*

*^c^ Section of Microbiology, Central Ayurveda Research Institute, Jhansi, CCRAS, Ministry of Ayush, Govt. of India*

*^d^ National Institute of Biologicals, Noida, Uttar Pradesh, India.*

*^e^ Amity Food and Agriculture Foundation, Amity University, Uttar Pradesh, India*

*^f^ Central Council for Research in Unani Medicine (CCRUM), Ministry of Ayush, Janakpuri, New Delhi, India*

**Author’s address:**

**Corresponding Author**:

**Prof. (Dr.) Kumud Bala**- Amity Institute of Biotechnology, Amity University, Uttar Pradesh, India, Sector 125, Noida, Uttar Pradesh 201313, India; Email: [kbala@amity.edu](mailto:kbala@amity.edu)


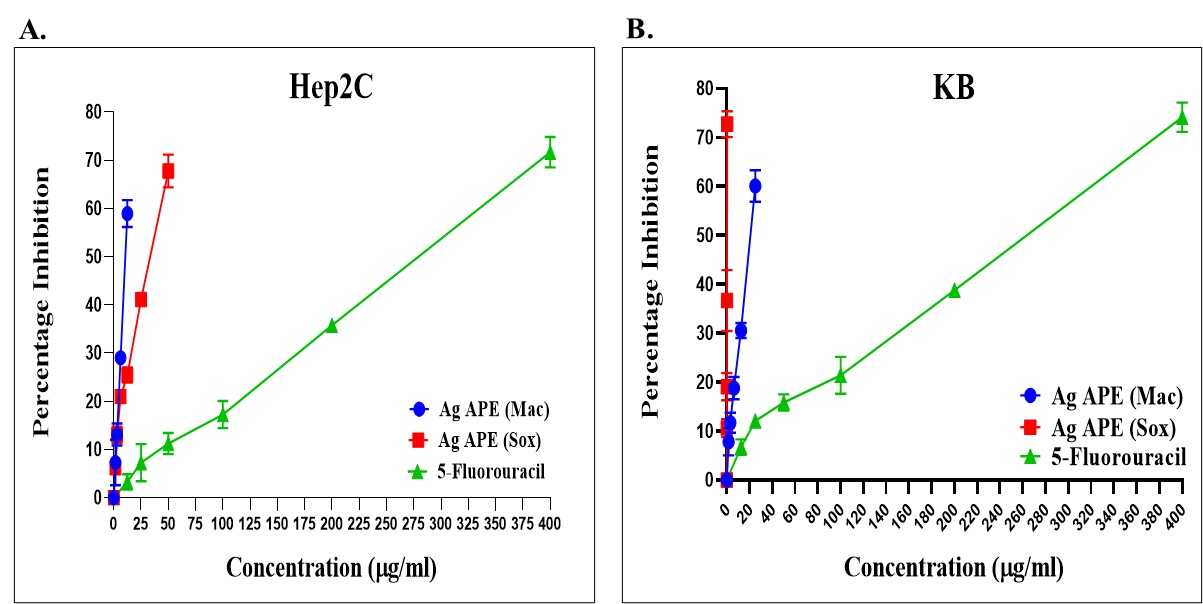


Graph 1: Growth inhibition curves on Hep2C and KB cell lines are determined by MTT assay for 48 h of treatment with; (**A)** 5- fluorouracil (12.5 - 400 μg/mL), AgAPE (Mac) (1.56 - 12.5 μg/mL) and AgAPE (Sox) (1.56 - 50 μg/mL); (**B**) 5-fluorouracil (12.5 – 400 μg/mL), AgAPE (Mac) (1.56 - 25 μg/mL) and AgAPE (Sox) (0.03 – 0.31 μg/mL). Data are shown as mean ± SD of three independent experiments.
